# Surgical management of postpartum haemorrhage: survey of French obstetricians

**DOI:** 10.1038/srep30342

**Published:** 2016-07-27

**Authors:** Pierre-Emmanuel Bouet, Stéphanie Brun, Hugo Madar, Elsa Schinkel, Benjamin Merlot, Loïc Sentilhes

**Affiliations:** 1 Department of Obstetrics and Gynecology, Montreal University Hospital—Montreal, Canada; 2 Department of Obstetrics and Gynecology, Bordeaux University Hospital—Bordeaux, France; 3 Clinical Research Center, Angers University Hospital—Angers, France

## Abstract

The aim of our study was to assess the theoretical and practical knowledge of French obstetricians about the surgical management of postpartum haemorrhage (PPH). Our study is a national anonymous self-administered survey. A total of 363 obstetricians responded to this questionnaire between December 2013 and April 2014. Questionnaire sent through email to all French obstetricians who are members of either of two federations of hospital-based obstetricians. Answers were collected until the end of June 2014. The main outcome measure was obstetricians’ level of mastery of each surgical technique. The results were analysed descriptively (proportions). Only the 286 questionnaires fully completed were analysed; the complete response rate was 23% (286/1246). In all, 33% (95/286) of the responding obstetricians reported that they had not mastered sufficiently or even at all the technique for bilateral ligation of the uterine arteries, 37% (105/286) for uterine compression suture, 62% (178/286) for ligation of the internal iliac arteries, and 47% (134/286) for emergency peripartum hysterectomy. In all, 18% (52/286) of respondents stated that they had not mastered any of these techniques. Our study shows that a worrisome number of French obstetricians reported insufficient mastery of the surgical techniques for PPH management.

Postpartum haemorrhage (PPH) remains the leading cause of maternal mortality both worldwide, causing 150 000 deaths a year, and in France, where it accounts for 15.5% of maternal deaths^1^. Approximately 1.7% of births are complicated by severe PPH (blood loss ≥ 1000 ml in the 24 hours after delivery)^2^. It appears that at least 80% of maternal deaths from PPH are avoidable, since they appear very often to follow a delay in diagnosis or management, or insufficient treatment^3^.

A recent observational study in France observed that arterial embolization is used in 2.7% (183/6660) of PPH, while uterine-sparing surgical procedures are used in 1.3% (86/6660) and hysterectomy in 1.1% (72/6660)^4^. A retrospective study in Ireland found that the prevalence of emergency hysterectomy after PPH was around 1/2000 deliveries^5^. Surgical treatment of PPH is used as a last resort when earlier procedures (prostaglandins, intrauterine tamponade, embolization) have failed, or sometimes from the start, when a severe life-threatening PPH requires that bleeding stop in the shortest time possible. All obstetricians can face these situations during their practice. They must therefore have the surgical skills to perform a hysterectomy, even if they work in a facility where they can seek help from a colleague on-call at home, or an abdominal surgeon on-call onsite. Failure to perform a surgical procedure necessary for PPH, most especially an emergency hysterectomy, or delay due to awaiting assistance from a colleague can lead to criminal and civil liability.

The principal objective of our study was to assess the theoretical and practical knowledge of French obstetricians about the surgical management of PPH. As secondary objectives, we sought to determine the surgical procedure that they would use as first, second, and third line treatments.

## Material and Methods

Our study is an anonymous survey, self-administered online. French obstetricians working in either the private or public sector and members of one of two federations of French hospital-based obstetricians (the federation of perinatal networks or the federation of circles of gynaecologists-obstetricians in non-university hospital centres) (n = 1248) received an explanatory email with a link to a survey containing 60 questions.

A first email was sent to each obstetrician in December 2013, and reminders were sent in February and in April, 2014. Only fully completed questionnaires were analysed. We considered a questionnaire fully completed when every question had a response (or several, when more than one choice was allowed). Answers were collected until the end of June 2014. Informed consent was obtained from all participants. The study protocol was approved by the Committee of Ethics and Research in Gynaecology-Obstetrics (CEROG-2014-08) and conducted in accordance with relevant guidelines.

The obstetricians worked in different types of maternity units. In France, these are classified in 3 levels: level III (full-time obstetric, paediatric, neonatal intensive care and anaesthesia staff) for premature births before 32 weeks of gestation, level II (full-time obstetric, paediatric and anaesthesia staff, and no long-term assisted ventilation) for premature births after 32 weeks of gestation and level I (no facilities for special care)^6^.

Because our research found no validated questionnaire about the topic of our survey in the literature, we — that is, all the co-authors — designed our own questionnaire, which was approved by CEROG. Evidence supports the reliability of self-assessment of technical skills in surgery^7^.

Some questions were dichotomous, and others open-ended. The questionnaire was designed on the website of www.limesurvey.com; responses were forced for each question, skip logic and a progress bar were used, and data were collected via Web-link and email and downloaded to a spreadsheet.

Each email sent to the obstetricians included a cover letter informing them of the time we estimated it would take them to complete the questionnaire (10–15 minutes) and stating that participation was voluntary, anonymous and unremunerated. An internet link enabled the physicians to accede to the questionnaire and complete it online. Unique identifiers were assigned to each participant’s computer, thereby ensuring that each individual could complete only one questionnaire.

The final questionnaire (Appendix) contained 3 sections. The first comprised demographic and institutional questions (n = 21). The second focused on the participants’ theoretical and practical knowledge (36 questions). The third section included questions about the sequence of techniques that they would use for surgical management of PPH for a young woman who wanted more children, was haemodynamically stable and managed by a multidisciplinary team (surgeon and anaesthesiologist) (3 questions).

The different surgical techniques mentioned in the questionnaire were: uterine compression sutures (UCS), including the B-Lynch and Cho suturing techniques^8^,^9^, bilateral uterine artery ligation (UAL)^10^, triple uterine artery ligation (TUAL)^11^,^12^, peripartum hysterectomy (PH)^13^, bilateral internal iliac artery ligation (IIAL), and stepwise uterine devascularisation (SUD)^14^,^15^,^16^.

The principal endpoint of the study was the obstetricians’ self-assessed level of mastery of each surgical technique that might be performed during the management of severe PPH. The physicians were asked for each technique whether they considered that they had mastered it completely, sufficiently, insufficiently, or not at all (questions 27, 33, 39, 45, 51 and 57).

As secondary outcome measures, we asked participants what surgical technique they would use as first, second, and third line treatments for severe PPH (questions 58–60).

Descriptive statistics were used to detail demographic characteristics, theoretical and practical knowledge, and the different management strategies employed by survey participants. For the quantitative data, comparisons were performed with the Mann-Whitney-Wilcoxon test for independent samples. Proportions were compared with the chi-square test. We used SPSS software, version 15.0 for Windows, SPSS Inc., Chicago, IL, USA. Statistical significance was defined as a *P* value < 0.05.

## Results

When we take incomplete as well as unreturned questionnaires into account, the participation rate was 23% (286/1246); there were 286 complete responses. The 77 questionnaires not fully completed were excluded from the study.

Table 1 summarises the participants’ demographic data. Their median age was 45 years (range: 35–55). The mean age of hospital staff physicians was not significantly different from that of the physicians working exclusively in the private sector (respectively, 45.9+/−4.6 and 46.0+/4.7; P = 0.9). The mean time in practice since the end of their residencies was 15.5+/−11.7 years. Most (78%, 224/286) worked in the public sector; 57% (164/286) were hospital staff physicians. Preferred subspecialties were obstetrics 55% (156/286), surgery 32% (93/286), prenatal diagnosis 9% (25/286) and reproductive medicine 4% (12/286). The median number of on-call duty sessions monthly was 4 (range: 3–5), and 46% (133/286) worked in level III units. Almost all (98%, 279/286) reported that they could always call a colleague if needed. Sixty percent (171/286) reported that their department had a written protocol for surgical management of severe PPH.

An embolization centre was available onsite for 51% (145/286) and a blood bank for 56% (160/286). On the other hand, 85 doctors (26%) worked more than 20 km from an embolization centre.

Surgical management of PPH was considered extremely stressful by 22% (63/286) and stressful by 66% (190/286).

Overall, 93% (267/286) of participants considered that they knew the theory of the UCS techniques, 98% (279/286) UAL, 89% (255/286) TUAL, 31% (89/286), SUD, 97% (278/286) IIAL and 99% (284/286) PH. On the other hand, 37% (105/286) reported they had not mastered the techniques of UCS, 33% (95/286) UAL, 40% (115/286) TUAL, 86% (247/286) SUD, 62% (178/286) IIAL, and 47% (134/286) PH. Moreover, 18% (52/286) reported that they had not mastered any of these techniques.

As Table 2 shows, 49% (140/286) had performed UCS alone, without help from a colleague, 57% (163/286) UAL, 47% (134/286) TUAL, 8% (22/286) SUD, 33% (93/286) IIAL, and 54% (154/286) a PH, while 27% (77/286) had never performed any of these procedures alone. In addition, 17.5% (50/286) had already performed UCS with a colleague, 17% (48/276) UAL, 15% (44/286) TUAL, 2% (6/286) SUD, 26% (74/286) IIAL and 34% (97/286) PH. At the same time, 26% (74/286), had never seen UCS performed, 31% (90/286) TUAL, or 24% (68/286) IIAL (Table 2).

These obstetricians considered that UCS could be completely mastered after performing 5 procedures (IQR: 3–10), UAL 4 (1–5), TUAL 5 (3–10), SUD 4 (1–5.5), IIAL 9 (3–20) and PH 8 (4–10). Table 3 reports these results as medians and their interquartile ranges. Comparing the median of the IIAL to that for UAL (9 vs 4), that for TUAL (9 vs 5) and finally that for UCS (9 vs 5) shows a significant difference in all 3 cases (*P* < 0.05).

In response to a hypothetical severe PPH requiring surgical management in a young, haemodynamically stable woman who wanted to be able to have more children, 51% (145/286) of the obstetricians said that their first line treatment would be a distal ligation technique (UAL, TUAL or SUD), 36% (103/286) UCS, 12% (35/286) IIAL and 1% (2/286) PH. Their second line choices were distal ligation (39%, 111/286), IIAL (32%, 90/286), UCS (17%, 50/286) and PH (11%, 32/286). Finally, their third-line choices were PH (55%, 156/286), IIAL (26%, 74/286), UCS (7%, 21/286) and distal ligation (1%, 3/286). Table 4 reports these results.

The surgical sequences used most often by the obstetricians were: (1) distal ligation (TUAL or UAL), then IIAL, and then PH for 22% of the participants, and (2) UCS, then distal ligation, then IIAL, and then PH for 18%.

## Discussion

Our study shows that most participants report that they know the theory of the different surgical techniques that can be used for severe PPH. On the other hand, a worrisome number of obstetricians (18%) reported they had not mastered any of these techniques, and 47% that they had not sufficiently (or not at all) mastered the technique of hysterectomy, the last-ditch treatment for severe PPH.

The high proportion of obstetricians with limited surgical skills for dealing with severe PPH may be the cause of the delayed management observed in deaths due to PPH in France^1^. Fortunately, 98% of the respondents reported that they could always call a colleague if they were having difficulty managing a case of severe PPH.

It is reassuring to note that 99% of participants report that their unit has a written protocol for medical management of PPH. Nonetheless, only 60% of participants mentioned the existence of a written protocol for its surgical management. Current French guidelines for PPH recommend that each obstetrical unit draft a full protocol for surgical management based on the local conditions and especially the environment quickly available in delivery room^17^.

Several hypotheses may explain the low level of mastery of this set of surgical techniques. First, the advent of uterine artery embolization in France has reduced recourse to haemostatic surgery in cases of severe PPH: embolization is used here at a rate at least double that of both uterine-sparing surgical procedures and hysterectomy^4^,^13^,^18^.

Second, developments in surgical technique and especially the emergence of laparoscopy for gynaecologic surgery may have contributed to reducing the practice and/or learning of laparotomic hysterectomies by obstetricians^19^, especially the youngest^20^.

Finally, early orientation during residency in gynaecology-obstetrics toward subspecialties such as prenatal diagnosis or reproductive medicine may be reducing the exposure of young obstetricians to the practice of laparotomic hysterectomies.

As a first line treatment, these obstetricians preferred the use of distal ligation techniques (51%) or uterine compression sutures (36%) to those for IIAL (12%). The learning curves for the first two appear to be significantly faster than that for the latter. Participants considered that they had mastered the distal ligation or uterine compression techniques after 4 or 5 procedures while it took them 9 to master IIAL. We have not found other studies in the literature comparing the perceptions of physicians about the number of procedures to be performed before mastery. On the other hand, several authors have already mentioned that IIAL is a difficult procedure, especially in emergencies; it requires real learning and practice and is not very reproducible, except perhaps by gynaecological surgeons specialised in oncology^21^,^22^.

Moreover, IIAL appears to be less effective than UAL or UCS techniques^13^,^14^,^21^. Doumouchtsis *et al*. have reviewed the literature about the effectiveness of ligation of the internal iliac arteries^23^. They report a mean effectiveness rate for IIAL of 69% compared with 93% for UAL and 83% for uterine compression^23^. No comparative study has yet demonstrated the superiority of one of these techniques compared with another. Accordingly there are no international guidelines proning the use of one technique of conservative surgery compared with another^24^,^25^.

Nonetheless, given the high number of physicians (18%) mastering none of these techniques for haemostatic surgery and the long learning time for IIAL, and considering that most obstetricians do not subspecialise in gynaecologic oncology surgery, it appears important to focus training on mastery of UAL, UCS and hysterectomy. Accordingly, our team^12^,^26^ uses UAL or uterine compression (but not IIAL) as the first line conservative choice when PPH is resistant to medical treatment.

No guidelines provide specific indications for postpartum hysterectomy^27^. The Royal College of Obstetricians and Gynaecologists (RCOG) recommend hysterectomy “sooner rather than later” and with the assistance of a second consultant^25^. French guidelines note that peripartum hysterectomy must be considered as the first line surgical treatment of massive PPH that is not responsive to earlier interventions or which is accompanied by cardiovascular instability^17^.

Different paedagogical tools are available to develop continuing medical education and to promote the learning of these techniques: videos of techniques performed in real time available on DVD^28^ or the internet^29^, the acquisition of different surgical techniques with the help of specific books^30^,^31^ or instructional charts^17^,^21^ or participation in simulation workshops^32^, such as those proposed by the RCOG^33^.

It is important that obstetricians be aware of the various learning difficulties. Despite the presence of different pedagogical tools, experienced obstetricians mastering the complex surgical techniques should take part in promoting and teaching these skills among their junior and less experienced colleagues. Moreover, they could assist their fellow senior obstetricians less at ease with these techniques in order to expedite their learning^34^.

On the other hand, the national Obstetrics and Gynecology residency training program should include special training modules on the surgical management of PPH, using all the pedagogical tools previously mentioned. Finally, considering the relative rare occurrence of PPH in everyday practice, workshops and seminars on the surgical management should be part of the continuous medical education programs in the annual congresses and meetings attended by obstetricians.

This study has significant strengths and is, to our knowledge, the first to assess obstetricians’ theoretical and practical knowledge about the surgical management of PPH. Data came from a large survey with a diverse range of participants from throughout France. Our study has several limitations: it was a self-reported survey with a low response rate and what might be viewed as limited generalizability. In particular, respondents in private practice are probably underrepresented^35^. In France, 35% of deliveries take place in the private sector while 12% of participants reported working solely in this sector and 10% working both in the private and public sectors^35^. Most respondents (57%) reported that they worked in the public sector; the mean age of these two groups was, however, similar.

In conclusion, our survey showed that 18% of responding obstetricians working onsite on-call duty at hospitals reported that they had not mastered any of the techniques of emergency obstetric surgery. Moreover, 47% reported that they had not mastered sufficiently (or at all) the technique of hysterectomy, which is the essential procedure for maternal salvage in severe PPH resistant to medical treatment. Our results suggest there are extreme deficiencies in the training of modern day obstetricians in France, and that adjustments in the national residency training program are required to address these weaknesses. It might be useful for other countries to perform a similar survey to help determine if these alarming results are particular to France.

## Additional Information

**How to cite this article**: Bouet, P.-E. *et al*. Surgical management of postpartum haemorrhage: survey of French obstetricians. *Sci. Rep*. **6**, 30342; doi: 10.1038/srep30342 (2016).

## Supplementary Material

Supplementary Information

## Figures and Tables

**Table 1:** Characteristics of participating obstetricians and the maternity units in which they worked.

	Total n = 286
Sex	
• Female	100 (35; 29–41)
• Male	186 (65; 59–71)
Age (years)^1^	45 (35–55)
Years in practice	15.5 + /− 11.7
Sector	
• Public	224 (78; 73–83)
• Private	34 (12; 8–16)
• Public/private	28 (10; 7–14)
Status	
• Assistant Chief Resident/Chief Resident	44 (15; 11–20)
• Hospital staff physician	164 (57; 51–63)
• University Professor - Hospital staff physician	26 (9; 6–13)
• Physician from abroad	4 (2; 1–3)
• Physician working in the private sector	31 (11; 7–15)
• Prefers not to say	17 (6; 3–9)
Preferred subspecialty	
• Surgery	93 (32; 27–38)
• Obstetrics	156 (55; 49–60)
• Prenatal diagnosis	25 (9; 6–13)
• Assisted reproduction	12 (4; 2–7)
Number of on-call duty shifts per month^1^	4 (3–5)
Level of maternity unit in which they work	
• I	54 (19; 14–24)
• II	99 (35; 29–40)
• III	133 (46; 40–52)
Number of deliveries/year in the maternity unit in which they work	2627 + /− 1329
Obstetrician on-call onsite	
• Yes	236 (83; 78–87)
• No	50 (17; 13–22)
Obstetrician on-call at home as well as obstetrician on-call onsite	
• Yes	129 (45; 38–51)
• No	157 (55; 49–61)
Can always call a colleague for help?	
• Yes	279 (98; 95–99)
• No	7 (2; 1–5)
Department has a written protocol for medical management of postpartum haemorrhage?	
• Yes	284 (99; 97–100)
• No	2 (1; 0–2)
Department has a written protocol for surgical management of postpartum haemorrhage?	
• Yes	171 (60; 54–66)
• No	115 (40; 34–66)
Site of the closest embolization centre	
• In hospital	145 (51; 45–57)
•<20 km from hospital	66 (23; 18–28)
• From 20 to 50 km from hospital	23 (8; 5–11)
•>50 km from your hospital	62 (18; 14–23)

Data are expressed as means + /− standard deviations or n (%; 95% confidence intervals).

^1^Median (interquartile range).

**Table 2:** Obstetricians’ practical knowledge.

	Performed alone^1^	Performed with help from a fully qualified doctor^1^	Seen it performed^1^	Never seen it performed^1^
Uterine compression sutures	140 (49; 43–55)	50 (17.5; 13–22)	47 (16; 12–21)	74 (26; 21–31)
Bilateral ligation of the uterine arteries	163 (57; 51–63)	48 (17; 13–21)	56 (20; 15–25)	43 (15; 11–20)
Tsirulnikov’s triple ligation	134 (47; 41–53)	44 (15; 11–20)	40 (14; 0.5–19)	90 (31; 26–37)
Stepwise uterine devascularisation	22 (8; 5–11)	6 (2; 1–4.5)	8 (3; 1–5)	251 (88; 83–91)
Bilateral ligation of the internal iliac arteries	93 (33; 27–38)	74 (26; 21–31)	73 (25.5; 21–30)	68 (24; 19–29)
Emergency hysterectomy	154 (54; 48–60)	97 (34; 28–40)	53 (18.5; 14–23.5)	17 (6; 3.5–9)

The data are reported in n (%; 95% confidence intervals).

^1^Participating obstetricians reported the number of times they were confronted with each situation for each of the mentioned techniques. For each technique, n corresponds to the number of participants who responded “Yes” to the various situations described.

**Table 3:** Median number of techniques completely or sufficiently mastered, calculated based on the number of procedures performed by the obstetrician, either alone or with the help of a colleague.

Level of mastery Techniques	Completely	Sufficiently
Uterine compression sutures	5 (3–10)	2 (0–3)
Ligation of uterine arteries	4 (1–5)	2 (0–4)
Tsirulnikov’s triple ligation	5 (3–10)	2 (0–3)
Stepwise uterine devascularisation	4 (1–5,5)	0 (0–2)
Ligation of the internal iliac arteries	9 (3–20)	3 (0–5)
Emergency hysterectomy	8 (4–10)	3 (2–5)

The data are reported as medians (and their standard deviations).

**Table 4:** Surgical technique reported to be used by obstetricians as first, second and third line treatments in a haemodynamically stable patient during severe PPH.

	First line	Second line	Third line
Uterine compression techniques	103 (36; 30–42)	50 (17; 13–22)	21 (7; 5–11)
Distal ligation (of the uterine arteries or Tsirulnikov stepwise triple ligation)	145 (51; 45–57)	111 (39; 33–45)	3 (1; 0–3)
Bilateral ligation of the internal iliac arteries	35 (12; 9–17)	90 (32; 26–37)	74 (26; 21–31)
Emergency hysterectomy	2 (1; 0–3)	32 (11; 8–15)	156 (55; 49–60)
None, a hysterectomy was already done	—	2 (1; 0–3)	32 (11; 8–15)

The data are expressed as n (%; 95% confidence intervals).
