# Supplementary material for: Surgical management of postpartum haemorrhage: survey of French obstetricians
Source: Sci Rep. 2016 Jul 27;6:30342. doi: 10.1038/srep30342 (PMC4962082; doi:10.1038/srep30342)
Supplement: Supplementary Information [file srep30342-s1.pdf]

Surgical management of postpartum haemorrhage: survey of French obstetricians

Pierre-Emmanuel Bouet, M.D., Stéphanie Brun, M.D., Hugo Madar, M.D., Elsa Schinkel, M.D., Benjamin Merlot, M.D., Loïc Sentilhes, M.D., Ph.D.

Appendix. Questionnaire about the surgical management of severe postpartum haemorrhage.

1 – What is your sex?

- Male
- Female

2 – How old are you?

3 – How many years have you been in practice since you became fully qualified?

4 - Activity Sector?

- Public (University or Regional Hospital Centre)
- Private
- Public-Private

5 – What is your status?

- Assistant Chief Resident/Chief Resident
- Hospital staff physician
- University Professor - staff physician
- Foreign physician
- Physician working in the private sector

6 – What is your degree?

- Diploma of Specialised Studies in Gynaecology-obstetrics
- Other Diploma of Specialised Studies
- Certificate of Specialised Studies

- Hospital staff physician equivalence, by a physician from abroad

7 – What is your preferred subspecialty?

- Gynaecologic surgery
- Obstetrics
- Ultrasound-prenatal diagnosis
- Assisted reproductive technology

8 – What is the level of the maternity unit where you currently practice?

- Level I
- Level II
- Level III

9 – Approximately how many deliveries a year are there at this maternity unit?

10 – How many times are you on-call onsite monthly, on average?

11 – Is there routinely a fully qualified gynaecologist-obstetrician present onsite (24/7) while you are on call?

- Yes
- No

12 - Is there a doctor on-call at home in addition to the senior obstetrician onsite?

- Yes
- No

13 – What is the approximate annual caesarean rate in the maternity unit where you work?

14 - How far from the hospital where you work is the nearest embolisation centre (in km)?

- In-hospital embolisation centre
- Embolisation centre at another hospital < 20 km away
- Embolisation centre at another hospital 20-50 km away
- Embolisation centre at another hospital > 50 km away

15 – Where is the blood bank in relation to your hospital?

- In hospital (onsite)
- < 20 km away
- 20-50 km away
- < 50 km away

16 - Does your department have a written protocol for the <u>medical</u> management of PPH in the delivery room?

- Yes
- No

17 - Does your department have a written protocol for the <u>surgical</u> management of PPH in the delivery room?

- Yes
- No

18 – In your written protocol for management of severe PPH, is the possibility of calling a colleague explicitly mentioned?

- Yes
- No
- No protocol for management of severe PPH in the delivery room.

19 – In practice, can you always call a colleague if you need one in cases of PPH (physician on-call at home, department head, other obstetrician, other abdominal surgeon)?

- Yes
- No

20 – Do you consider that the situation of severe PPH requiring surgical treatment is:

- extremely stressful?
- Stressful?

- a little stressful?
- absolutely not stressful?

21 – Do you know the protocol for medical management of postpartum haemorrhage in the delivery room ?

- Yes
- No

22 – Do you know the theory of uterine compression suture techniques (B-Lynch or Cho)?

- Yes
- No

23 - In practice, have you (choose all the responses that apply to you)

- performed it alone?
- performed with a fully qualified colleague?
- seen it performed?
- never seen it performed?

24 – How many times have you performed it alone? (answer only if you answered question 23: "performed it alone”)

25 - How many times have you performed it aided by a colleague? (answer only if you answered question 23: "performed with a qualified colleague")

26 – How many times have you seen it performed? (answer only if you answered question 23: "seen it performed")

27 – Do you consider that you have mastered this surgical technique

- completely?
- sufficiently?
- insufficiently?
- not at all?

28 – Do you know the surgical technique for bilateral ligation of the uterine arteries?

- Yes
- No

29 - In practice, have you (choose all the responses that apply to you)

- performed it alone?
- performed with a fully qualified colleague?
- seen it performed?
- never seen it performed?

30 – How many times have you performed it alone? (answer only if you answered question 29: "performed it alone")

31 - How many times have your performed it aided by a colleague? (answer only if you answered question 29: "performed with a qualified colleague")

32 – How many times have you seen it performed? (answer only if you answered question 29: "seen it performed")

33 – Do you consider that you have mastered this surgical technique

- completely?
- sufficiently?
- insufficiently?
- not at all?

34 – Do you know the theory of the Tsirulnikov triple ligation technique?

- Yes
- No

35 - In practice, have you (choose all the responses that apply to you)

- performed it alone?
- performed with a fully qualified colleague?

- seen it performed?
- never seen it performed?

36 – How many times have you performed it alone? (answer only if you answered question 35: "performed it alone")

37 - How many times have you performed it aided by a colleague? (answer only if you answered question 35: "performed with a qualified colleague")

38 – How many times have you seen it performed? (answer only if you answered question 35: "seen it performed")

39 – Do you consider that you have mastered this surgical technique

- completely?
- sufficiently?
- insufficiently?
- not at all?

40 - Do you know the theory of the stepwise uterine devascularisation technique described by AbdRabbo?

- Yes
- No

41 - In practice, have you (choose all the responses that apply to you)

- performed it alone?
- performed it with a fully qualified colleague?
- seen it performed?
- never seen it performed?

42 – How many times have you performed it alone? (answer only if you answered question 41: "performed it alone")

43 - How many times have you performed it aided by a colleague? (answer only if you answered question 41: "performed with qualified colleague")

44 – How many times have you seen it performed? (answer only if you answered question 41: "seen it performed")

45 – Do you consider that you have mastered this surgical technique

- completely?
- sufficiently?
- insufficiently?
- not at all?

46 – Do you know the theory for the surgical technique of ligation of the uterine arteries?

- Yes
- No

47 - In practice, have you (choose all the responses that apply to you)

- performed it alone?
- performed with a fully qualified colleague?
- seen it performed?
- never seen it performed?

48 – How many times have you performed it alone? (answer only if you answered question 47: "performed it alone)

49 - How many times have you performed it aided by a colleague? (answer only if you answered question 47: "performed with a qualified colleague")

50 – How many times have you seen it performed? (answer only if you answered question 47: "seen it performed")

51 – Do you consider that you have mastered this surgical technique

- completely?
- sufficiently?
- insufficiently?
- not at all?

52- Do you know the theory for the technique for emergency hysterectomy?

- Yes
- No

53 - In practice, have you (choose all the responses that apply to you)

- performed it alone?
- performed with a fully qualified colleague?
- seen it performed?
- never seen it performed?

54 – How many times have you performed it alone? (answer only if you answered question 53: "performed it alone")

55 - How many times have your performed it aided by a colleague? (answer only if you answered question 53: "performed with a qualified colleague")

56 – How many times have you seen it performed? (answer only if you answered question 53: "seen it performed")

57 – Do you consider that you have mastered this surgical technique

- completely?
- sufficiently?
- insufficiently?
- not at all?

58 – In practice, what surgical technique would you use as first line treatment? Consider the situation of a young woman, haemodynamically stable, desirous of more children, managed by a multidisciplinary team (surgeon and anaesthesiologist):

- Ligation of the uterine arteries or Tsirulnikov triple ligation or stepwise uterine devascularisation.
- Ligation of the internal iliac arteries.
- Uterine compression suture.
- Hysterectomy.

59 – If that failed, what surgical technique would you use as a second line treatment? Consider the situation of a young woman, haemodynamically stable, desirous of more children, managed by a multidisciplinary team (surgeon and anaesthesiologist)

- Ligation of the uterine arteries or Tsirulnikov triple ligation or stepwise uterine devascularisation.
- Ligation of the internal iliac arteries.
- Uterine compression sutures.
- Hysterectomy.
- None, a hysterectomy was already done.

60 – If that failed, what surgical technique would you use as a second line treatment? Consider the situation of a young woman, haemodynamically stable, desirous of more children, managed by a multidisciplinary team (surgeon and anaesthesiologist)

- Ligation of the uterine arteries or Tsirulnikov triple ligation or stepwise uterine devascularisation.
- Ligation of the internal iliac arteries.
- Uterine compression suture.
- Hysterectomy.

- None, a hysterectomy was already done.
